# Chronically Elevated Exogenous Glucose Elicits Antipodal Effects on the Proteome Signature of Differentiating Human iPSC-Derived Pancreatic Progenitors

**DOI:** 10.3390/ijms22073698

**Published:** 2021-04-02

**Authors:** Luiza Ghila, Thomas Aga Legøy, Andreas Frøslev Mathisen, Shadab Abadpour, Joao A. Paulo, Hanne Scholz, Helge Ræder, Simona Chera

**Affiliations:** 1 Center for Diabetes Research, Department of Clinical Science, Faculty of Medicine, University of Bergen, 5021 Bergen, Norway; Thomas.Legoy@uib.no (T.A.L.); Andreas.F.Mathisen@uib.no (A.F.M.); Helge.Rader@uib.no (H.R.); 2 Hybrid Technology Hub-Centre of Excellence, Faculty of Medicine, University of Oslo, 0318 Oslo, Norway; Shadab.Abadpour@rr-research.no (S.A.); hanne.scholz@medisin.uio.no (H.S.); 3 Institute for Surgical Research and Department of Transplant Medicine, Oslo University Hospital, 0027 Oslo, Norway; 4 Department of Cell Biology, Harvard Medical School, Boston, MA 02115, USA; Joao_Paulo@hms.harvard.edu; 5 Department of Pediatrics, Haukeland University Hospital, 5021 Bergen, Norway; 6 Division of Endocrinology, Diabetes, Nutrition, Department of Medicine, Faculty of Medicine, University of Geneva, 1211 Geneva, Switzerland

**Keywords:** cell identity, cell fate, in vitro differentiation, pancreatic endocrine progenitors, hiPSC, signaling pathway analyses, exogenous glucose, proteomics

## Abstract

The past decade revealed that cell identity changes, such as dedifferentiation or transdifferentiation, accompany the insulin-producing β-cell decay in most diabetes conditions. Mapping and controlling the mechanisms governing these processes is, thus, extremely valuable for managing the disease progression. Extracellular glucose is known to influence cell identity by impacting the redox balance. Here, we use global proteomics and pathway analysis to map the response of differentiating human pancreatic progenitors to chronically increased in vitro glucose levels. We show that exogenous high glucose levels impact different protein subsets in a concentration-dependent manner. In contrast, regardless of concentration, glucose elicits an antipodal effect on the proteome landscape, inducing both beneficial and detrimental changes in regard to achieving the desired islet cell fingerprint. Furthermore, we identified that only a subgroup of these effects and pathways are regulated by changes in redox balance. Our study highlights a complex effect of exogenous glucose on differentiating pancreas progenitors characterized by a distinct proteome signature.

## 1. Introduction

A shared feature of most diabetes disorders is the ultimate loss of functional insulin-producing pancreatic β-cells. This apparent common denominator hides an unexpected level of complexity: loss does not necessarily equal death. Although death might represent the final outcome, current research suggests a much more complex situation [1], where β-cells display a variety of initial responses which prevent or precede permanent loss, such as dedifferentiation [2,3] or transdifferentiation [4,5,6,7]. Thus, understanding the mechanisms governing identity maintenance in the pancreatic islet will allow for impeding or maybe even reversing the decay process.

Previous studies on a wide range of cell types indicated extracellular glucose concentrations as being a key contributor to cell fate decisions [8]. The impact of glucose levels is mainly attained through modulating the redox balance of the exposed tissue or cell type. The resultant reactive oxygen species act as secondary messengers improving differentiation and tissue remodeling. Nevertheless, if the redox disequilibrium is important or persistent, the oxidative stress will cause cellular damage and will ultimately result in apoptosis of the affected cell [8,9]. Consequently, the cellular antioxidant and repair mechanisms are of utmost importance for cell functionality and identity.

Pancreatic islet cells are no exception, with studies showing that short exposure to high glucose concentrations is able to potentiate β-cell function [10,11], while the physiological glucose concentration is critical for β-cell identity maintenance (reviewed in [12]). In contrast, long-term high glucose concentrations were clearly linked to β-cell dedifferentiation and dysfunction in different rodent models [2,12,13,14], cell lines [15] and human islets in vitro [16,17].

Generating islet cells and especially β-cells through guided in vitro differentiation from human-induced pluripotent stem cell (hiPSC) or embryonic stem cell (ESC) sources has a huge therapeutic potential [18,19,20,21], besides providing a context for demultiplexing the factors involved in β-cell fate acquisition, maintenance and pathology [22]. In the current differentiation protocols, hiPSCs/ESCs are differentiated in a stepwise manner, mimicking the natural stages of β-cell development [23,24,25]. Of importance, most published protocols incorporate a glucose concentration switch during the last differentiation stages (initiated at the pancreas progenitor stage), involving a raise from physiological (10 mM) to elevated (20 mM) glucose levels [24,26,27,28]. Consequently, the in vitro generated pancreatic progenitors gradually mature in a non-physiological hyperglycemic environment, which is, however, important for their differentiation.

We recently assessed the effect of short-term and long-term in vivo elevated hyperglycemia on encapsulated and xenotransplanted hiPSC-derived pancreatic progenitors. Of note, encapsulation increased the yield of islet cell types, without, however, improving cell identity selection [29], a parameter resolved by xenotransplantation into normoglycemic animals [30]. This beneficial effect on β-cell identity is lost when cells are transplanted into overly diabetic hosts due to hyperglycemia-induced oxidative stress, which results in the accumulation of immature cells and, following prolonged exposure, apoptosis [31]. It is, however, still unclear if exposure to elevated glucose concentrations elicit the same effects in vitro during the last stages of guided differentiation. Here, we used global proteomics to map the proteome landscape changes of differentiating pancreas progenitors in response to increased glucose concentrations in vitro. We show that, according to concentration, elevated exogenous glucose levels mobilize different metabolic and developmental responses. Moreover, by performing a comparative pathway analysis on glucose-, oxidant- and antioxidant-treated samples, we show that these effects are only partially relayed by changes in the redox balance and energy metabolism.

## 2. Results

### 2.1. High Glucose Concentrations Dysregulate Key Factors with Role in Pancreatic Islet Cells Development

In order to characterize the effects of in vitro hyperglycemia on the proteome fingerprint of differentiating islet cells, we exposed hiPSC-derived pancreatic progenitors to highly elevated glucose concentrations during the last two stages of differentiation. For hiPSC differentiation, we employed one of the most reliable and widely used protocols, elaborated by [24], which consists of seven differentiation steps over a period of one month. Of note, this protocol involves, by default, exposing the cells to a non-physiological glucose concentration (20 mM) from the pancreatic progenitor stage onwards (2 weeks, from stages 5 to 7) [24].

**Figure 1:**
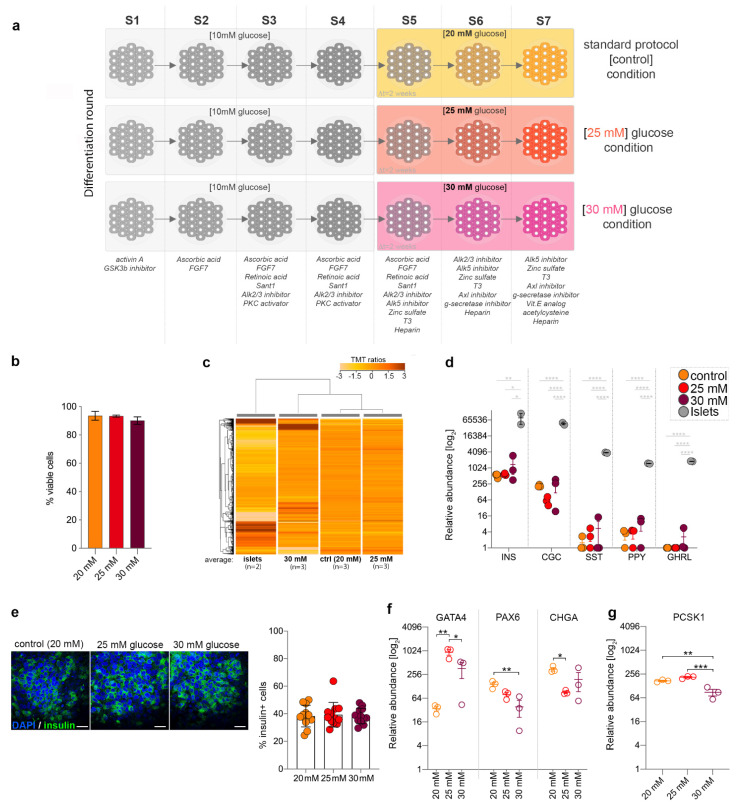
Elevated glucose levels’ effect on key pancreatic islet markers’ abundance. (**a**) Scheme depicting the glucose exposure conditions analyzed. (**b**) Cell viability at the end of stage 7 for the glucose exposure conditions. (**c**) Hierarchical clustering of the average tandem mass tag (TMT) ratios (*n* = 2,3,3,3). (**d**) Relative abundance levels of main islet hormones between the compared conditions. (**e**) Confocal imaging and quantification of insulin (Ins) + cells (green) in control (20 mM glucose) and samples exposed to either 25 or 30 mM ectopic glucose (Scale bar: 25 µm; blue–nuclear staining 4′,6-diamidino-2-phenylindole (DAPI). (**f**) Selected markers involved in pancreas development and (**g**) Proprotein convertase 1 (PCSK1) marker in standard (20 mM), 25 and 30 mM glucose conditions. Graphs are shown as mean ± SEM. Statistics: one-way ANOVA with Tukey’s multiple comparisons test. * *p* < 0.05, ** *p* < 0.01, *** *p* < 0.001, **** *p* < 0.0001.

Briefly, three distinct hiPSC colonies were differentiated during three distinct differentiating rounds as previously described [32,33]. Starting at the pancreatic progenitor stage (stage 5), cells were exposed to the appropriate differentiation cocktail containing either the protocol “standard” 20 mM glucose (standardly elevated) or higher glucose levels of 25 (mildly increased) and 30 mM (highly increased) (Figure 1a). Of note, the term “standard” will be further used to designate the standard, but not physiological, concentration required in the differentiation protocol and does not refer to “standard physiological conditions”.

Cell viability was found to be above 85% for all samples (Figure 1b). Subsequently, the different glycemic conditions as well as native human islets isolated from deceased donors were compared using global proteomics (tandem mass tag (TMT) 11-plex proteomics). The assay revealed 8858 unique proteins, with 7965 being detected in all conditions.

The hierarchical clustering of the averaged TMT ratios of 4947 differentially expressed proteins between stage 7 cells and human islets revealed the highest glucose concentration to have the strongest impact on the proteome landscape (Figure 1c), with the samples exposed to 30 mM glucose clustering farther away from the standard control (20 mM glucose).

We further interrogated the abundance of the main islet-specific hormones in the different conditions analyzed. There was no significant difference in the hormone levels between the samples differentiated by various glucose concentrations (Figure 1d,e). Markers with a key role in islet cells’ development such as GATA4 or chromogranin A (CHGA) were significantly dysregulated in the 25-mM samples (Figure 1f), while Paired Box 6 (PAX6) was impacted at the higher 30-mM concentration (Figure 1f), similar to the β-cell maturity maker PCSK1 (Figure 1g). Of note, the samples exposed to the higher glucose concentration displayed a high overall inter-replicon variation, which could account for fewer markers passing the significance threshold in this condition. These results suggest that increased glucose concentrations impact islet cell fate acquisition, although without a significant impact on hormone expression, besides glucagon (GCG).

### 2.2. Mildly Increased Glucose Levels Impact the Growth and Developmental Profile of the In Vitro Differentiating Cells

**Figure 2:**
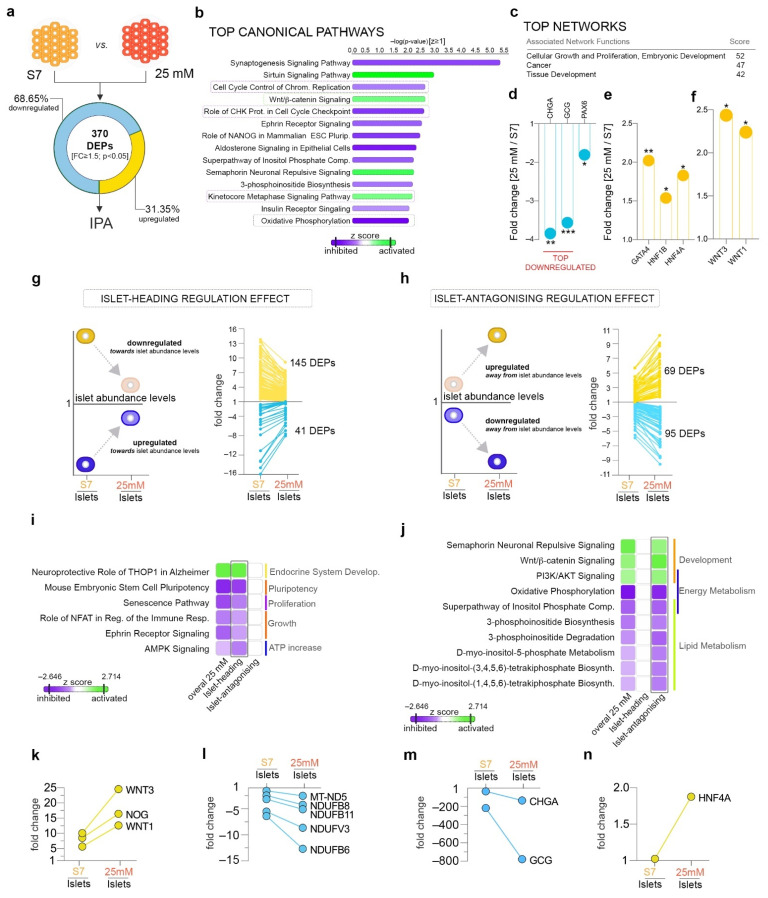
Pathway analysis of 25 mM glucose concentration effect. (**a**) Analysis workflow depicting the comparison employed. (**b**) Top canonical pathways with predicted regulation and (**c**) top networks characterizing the 25 mM glucose condition. (**d**) Selected dysregulated islet markers. (**e**) Selected differentially expressed developmental markers of pancreas development. (**f**) Graph of the observed downregulated WNT1 and WNT3 ligands in 25 mM glucose condition. (**g**) Scheme depicting the selection strategy and number of proteins displaying a dynamic of regulations compatible with an islet-heading or (**h**) an islet-antagonizing regulatory pattern in response to 25 mM glucose. Arrows represent the generic mandatory direction of regulation for inclusion in each respective subgroup. (**i**,**j**) Ingenuity Pathway Analysis (IPA)-generated comparison analysis of the protein landscapes characterizing the overall 25 mM effect, islet-heading and islet-antagonizing regulatory subgroups. (**k**–**n**) Graphs showing representative markers following an islet-antagonizing regulation. * *p* < 0.05, ** *p* < 0.01, *** *p* < 0.001.

To comprehensively characterize the effect of mildly increased glucose concentrations on differentiating islet cells, we performed a pathway analysis on the differentially expressed proteins (DEPs) between the cells exposed to 25 mM glucose and their controls (20 mM glucose). We detected 370 proteins being differentially expressed (Supplemental Table S1) in 25-mM-exposed samples as compared to the control, with ~2/3 (68.65%, 254/370) being downregulated and ~1/3 (31.35%, 116/370) being upregulated (Figure 2a). Moreover, just one of 14 previously characterized β-cell constitutively expressed proteins (Vesicle Associated Membrane Protein 2, VAMP2) [34] was found differentially expressed (Supplemental Table S2). Ingenuity Pathway Analysis software (IPA) revealed the regulation of pathways involved in cell cycle, energy metabolism, neurogenesis and development, amongst others (Figure 2b).

Briefly, pathways involved in neuro- and neuritogenesis were predicted as inhibited, with several being found in the top regulated pathways of the analyzed proteome landscape (Figure 2b). These results were corroborated by the disease and function analysis, which inferred the decrease in the neurodevelopment-related processes with a high level of confidence. Moreover, the dysregulation of this subset of proteins was the most representative for the global proteome landscape (Supplementary Figure S1a). These results suggest that mildly increased glucose levels inhibit pathways involved in neuronal development.

Furthermore, signaling involved in cell cycle check-points’ regulation was inferred as inhibited, while signaling related to mitotic progression was predicted as activated, suggesting dysregulated proliferation in 25-mM-exposed samples (Figure 2b, purple frames). In addition, oxidative phosphorylation signaling (OXPHOS, Figure 2b, black frame) was inhibited when compared to control samples, indicating a potential alteration of the energy metabolism. Last, the canonical Wnt pathway was indicated as activated in 25-mM-exposed samples, suggesting its potential impact on the differentiation potential.

Accordingly, the top-rated networks characterizing the analyzed regulatory landscape consisted of proteins involved in Cellular Growth and Proliferation, Cancer and Embryonic and Tissue Development (Figure 2c), further supporting the above conclusion of the impact of exposure to 25 mM glucose on proliferative, energy and developmental processes.

At single-protein resolution, the pan-endocrine marker chromogranin A (CHGA), GCG hormone and the PAX6 transcription factor were dysregulated, with the first two being the top downregulated proteins of the analyzed landscape (Figure 2d). Interestingly, several key factors involved in islet and β-cell development were significantly upregulated (Figure 2e) following mildly elevated glucose exposure, albeit without an obvious impact on hormone abundance, with the exception of glucagon. Unfortunately, due to the detection limitation of the techniques, we were unable to detect lowly expressed islet cell markers, such as pancreatic and duodenal homeobox 1 (PDX1), NKX6.1 (NK6 Homeobox 1), ARX (Aristaless Related Homeobox) or MAFA (musculoaponeurotic fibrosarcoma oncogene homolog). Beside key islet cell markers, WNT1 and WNT3 ligands were also found upregulated (Figure 2f), suggesting them as main activators of the canonical Wnt pathway in this context.

Overall, these results suggest an impact of the mildly elevated glucose levels on the in vitro proliferation potential and the differentiation program of the pancreatic progenitors, arguable through a Wnt-based mechanism.

### 2.3. Mildly Elevated Glucose Level Elicits both Beneficial and Detrimental Effects on the Islet Cell Signature of the Differentiating Cells

The above analysis provides insights into the general effects of mildly elevated glucose levels but fails to address their positive or negative impact on islet cell differentiation. Thus, to address which of the identified regulations are promoting an improved islet-like signature or, in contrast, inhibit it, we introduced islet samples for normalization. This allowed us to discriminate between the DEPs that change their abundance profiles in response to exposure to 25 mM glucose towards the levels detected in the islets (islet-heading regulation, Figure 2g) and the ones exhibiting the opposite regulation (islet-antagonizing regulation, Figure 2h). A total of 186 DEPs (fold change (FC) ≥ 1.5, *p* < 0.05) exhibited an islet-heading regulation, with the vast majority (77.96%, 145/181) being downregulated towards the islet abundance levels (Figure 2g). In contrast, 164 DEPs displayed (FC ≥ 1.5, *p* < 0.05) an islet-antagonizing regulation, being regulated away from the abundance levels normally detected in bona fide islets (Figure 2h).

The pathway comparison of these two subsets revealed the involvement of the proteins exhibiting islet-heading regulation in inhibiting pluripotency and growth, modulating proliferation and promoting endocrine system development and energy expenditure (Figure 2i, Supplementary Figure S1b). Of note, most of the top pathways were almost exclusively driven by the proteins belonging of this subset (i.e., proteins exhibiting islet-headed regulation, Figure 2i).

In contrast, the proteins displaying islet-antagonizing regulation are controlling developmental processes as well as energy and lipid metabolism (Figure 2j). Interestingly, key proteins driving the canonical Wnt signaling activation were included in this subset (selected examples in Figure 2k), suggesting an adverse impact of its activation on islet cell fate acquisition. Along the same line, the regulation away from islet abundance levels of the proteins controlling energy (complex I, Figure 2j,l, Supplementary Figure S1c) and lipid metabolism (Figure 2j, l, Supplementary Figure S1c) might also indicate that lipid synthesis inhibition and OXPHOS lowering represent harmful effects of the exposure to mildly elevated glucose concentrations.

As expected, GCG and CHGA were included in the islet-antagonizing effect due to their observed downregulation away from the islet abundance level in response to increased glucose levels (Figure 2m). Of interest, the HNF4A (Hepatocyte Nuclear Factor 4 Alpha) master regulator was significantly upregulated, arguably suggesting a potential indirect effect on glucagon levels (Figure 2n).

Taken together, these results suggest that mildly elevated glucose promotes differentiation and modulates proliferation towards islet-like regulation but impedes the achievement of a true islet profile by interfering with the energy and lipid metabolism as well as modifying the hormone expression patterns. Arguably, these effects are not entirely passive, as increased glucose levels negatively regulate signaling pathways with key developmental roles in differentiation.

### 2.4. Pathway Analysis Reveals a Largely Different Protein Regulation in Response to Highly Increased Glucose Levels

**Figure 3:**
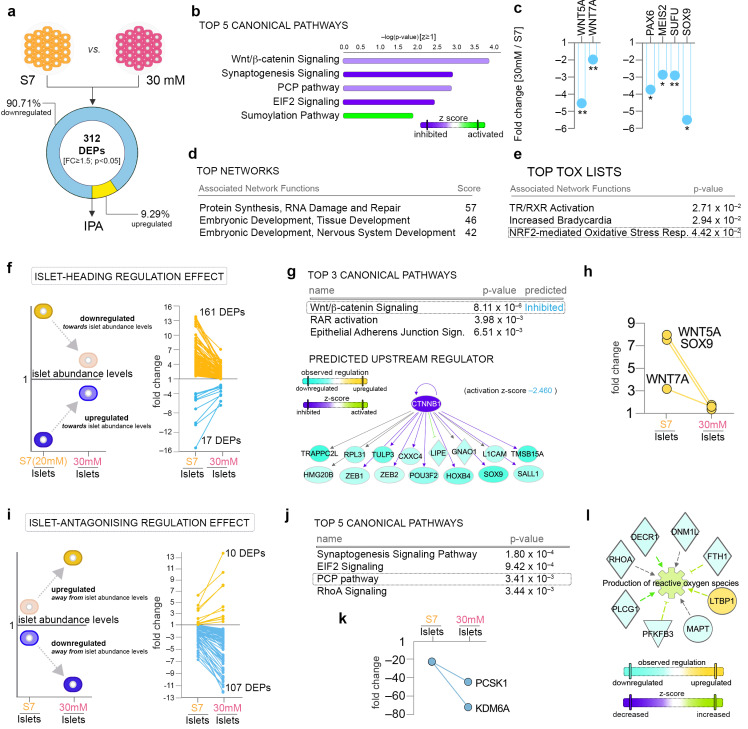
Pathway analysis of 30-mM glucose concentration effect. (**a**) Analysis workflow depicting the comparison employed. (**b**) Top canonical pathways with predicted regulation characterizing the 30-mM glucose condition. (**c**) Graphs of the observed downregulated WNT5A and WNT5B ligands and PAX6, MEIS2 (Meis homeobox 2), SUFU (suppressor of fused protein) and SOX9 (SRY-Box Transcription Factor 9) in the 30 mM glucose condition. (**d**) Top networks and (**e**) top tox list characterizing the proteome landscape of the 30 mM glucose condition. (**f**) Scheme depicting the selection strategy and number of proteins displaying a dynamic of regulations compatible with an islet-heading regulatory pattern in response to 30 mM glucose. (**g**) Top 3 canonical pathways and the network representation of one of the top predicted upstream regulators’ (Catenin Beta 1, CTNNB1) target molecules. (**h**) Graphs of WNT5A, WNT7A and SOX9 following an islet-heading regulation pattern. (**i**) Scheme depicting the selection strategy and number of proteins displaying a dynamic of regulations compatible with an islet-antagonizing regulatory pattern in response to 30 mM glucose. (**j**) Top 5 canonical pathways characterizing the subgroup of proteins displaying islet-antagonizing regulation. (**k**) Graphs of the β-cell markers PCSK1 and KDM6A (Lysine Demethylase 6A) presenting an islet-antagonizing regulation pattern. (**l**) IPA-generated network representations of selected dataset differentially expressed proteins (DEPs) characterizing the corresponding top disease and function processes. * *p* < 0.05, ** *p* < 0.01.

We further addressed whether the above proteome landscape regulation is retained when pancreatic progenitors are differentiated at higher glucose (30 mM) concentrations (Figure 3a). We identified 312 proteins being differentially expressed (FC ≥ 1.5, *p* < 0.05) between 30-mM-exposed samples and the control (20 mM glucose), with the vast majority of proteins being downregulated (90.71%, 283/312). The pathway analysis revealed the inhibition of canonical Wnt signaling in the top regulated pathways characterizing the global proteome landscape (Figure 3b), in stark contrast with its regulation following mildly elevated glucose (Figure 2b and Figure 3b). Along the same line, the PCP (planar cell polarity) pathway (non-canonical Wnt signaling) was also predicted as downregulated (Figure 3b, top 3), suggesting a global inhibition of Wnt signaling by the 30-mM glucose concentration. As expected, critical ligands and regulators of the canonical Wnt and PCP pathway, WNT5A and WNT7A, respectively, were significantly downregulated (Figure 3c).

In agreement with the unusually high number of downregulated DEPs, EIF2 (Eukaryotic Initiation Factor 2) signaling (inhibited) and sumoylation signaling (activated) were identified in the top five canonical pathways, indicating that exposure to 30 mM glucose negatively impacts protein synthesis and increases their turnover (Figure 3b). Accordingly, the top ranked network consisted of proteins involved in protein synthesis regulation, while the second and third top networks comprised of proteins involved in developmental processes (Figure 3d). At single-protein levels, key developmental transcription factors such as PAX6, MEIS2 and SOX9 and negative regulators such as SUFU were found significantly downregulated (Figure 3c).

Similar to the mildly elevated glucose concentration effect, the synaptogenesis signaling pathway was inferred as inhibited (Figure 3b), suggesting an inhibitory action of elevated glucose on the neurogenesis pathways regardless of concentration (25 or 30 mM).

Of note, the tox analysis of the 30-mM-exposed samples indicated interference with antioxidant mechanisms, such as the NRF2 (nuclear factor erythroid 2–related factor 2)-mediated oxidative stress response, an indicator of potential redox imbalance (Figure 3e).

Overall, these results suggest that the two distinct glucose concentrations elicit fairly different effects on differentiating pancreatic endocrine cells.

### 2.5. Highly Increased Glucose Level Modulates the Canonical Wnt Signaling towards Native Islet Regulation, while Negatively Impacting Energy Metabolism and Protein Synthesis

Following a similar strategy as above, we identified the subsets of proteins following an islet-heading regulatory pattern after 30-mM glucose exposure (Figure 3f). A total of 178 DEPs fulfilled this requirement, most being downregulated towards islet abundance levels (Supplemental Table S1). The pathway analysis of this subset revealed, in the top canonical pathway, canonical Wnt signaling inhibition (Figure 3g). In accordance, based on the observed regulatory landscape, the analysis predicted β-catenin as an upstream regulator. Accordingly, both WNT5A and WNT7A ligands as well as the SOX9 transcription regulator displayed an islet-heading regulation (Figure 3h). Of note, based on previous studies, 30 mM glucose is expected to elicit toxic effects on mature cells and tissues.

Furthermore, decreases in a wide range of processes related to neuronal development were indicated in the top disease and function (Supplementary Figure S2a), suggesting that inhibition of neurogenesis mechanisms by elevated glycemia levels potentially promotes islet cell fate. Moreover, the global integration of all pathway analysis levels is suggestive of a potential link between the inactivation of the canonical Wnt signaling pathway and decreased neurogenesis (Supplementary Figure S2b). Nevertheless, further experimental validation will be required to properly validate this observation.

In contrast, a subset of 117 DEPs displayed an islet-antagonizing regulation following exposure to high concentrations of glucose (Figure 3i). Interestingly, the pathway analysis showed inactivation of the EIF2 pathway (z-value = −2) as the second top canonical pathway (Figure 3j). Consistently, the network analysis indicated proteins involved in protein synthesis regulation in the top ranked network (Supplementary Figure S2c), suggesting the protein subset’s involvement in inhibiting protein synthesis. Moreover, integrin-based signaling (Ras Homolog Family Member A (RhoA) signaling, integrin signaling (2.25 × 10^−2^), actin cytoskeleton signaling (2.42 × 10^−2^)) and synaptogenesis signaling inhibition, as well as PCP pathways, also define the regulatory group (Figure 3j, Supplementary Figure S2c). These data promote the importance of cytoskeleton remodeling and non-canonical Wnt signaling activation during the last stages of differentiation.

At the single-protein level, both the neuroendocrine convertase PCSK1 and the key β-cell fate methylase KDM6A display an islet-antagonistic regulation, pointing towards differentiation and maturation problems of the islet cells as a consequence of high glucose exposure (Figure 3k).

Of interest, the disease and function analysis also indicated the increased production of reactive oxygen species in this context (Figure 3l), suggestive of potential oxidative stress damage.

### 2.6. The Differential Proteome Landscape between Mildly and Highly Increased Glucose Is Characterized by Reduced Protein Synthesis and Low Antioxidant Activity

**Figure 4:**
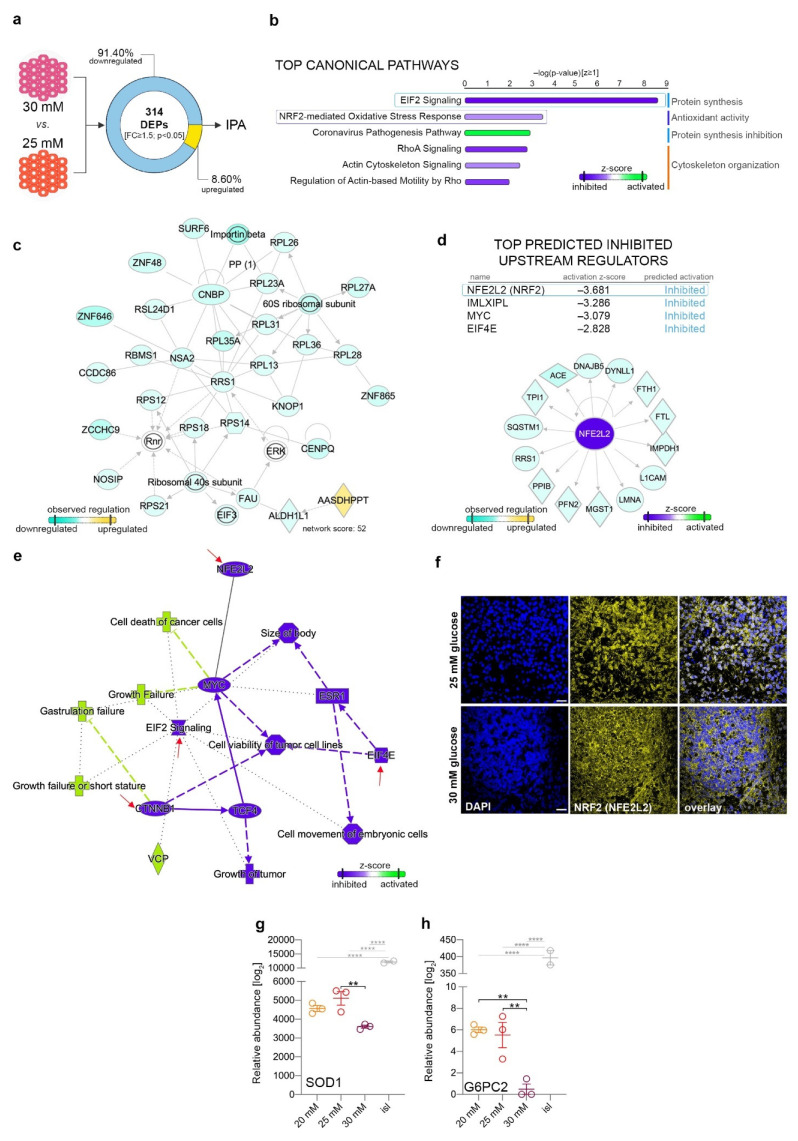
Direct comparison analysis between the proteome landscape of 30 and 25 mM glucose conditions. (**a**) Analysis workflow depicting the comparison employed. (**b**) Top canonical pathways with predicted regulation characterizing the differentially expressed protein landscape between 30 and 25 mM glucose conditions. (**c**) Selected top 2 organic networks displaying the observed downregulation of proteins involved in protein synthesis. (**d**) Top predicted activated upstream transcription regulators and the NFE2L2 (NRF2) target molecules observed regulated in the analyzed DEPs dataset. (**e**) IPA-generated global integration of all pathway analysis levels characterizing the differentially expressed protein landscape between 30 and 25 mM glucose conditions. (**f**) Confocal imaging of representative NRF2^+^ cells (yellow) in 25- and 30-mM_-_treated samples (Scale bar: 25 µm; blue–DAPI). (**g**) Relative abundance levels of superoxide dismutase 1 (SOD1) and (**h**) G6PC2 (Glucose-6-Phosphatase Catalytic Subunit 2) proteins (one-way ANOVA with Tukey’s multiple comparisons test, ** *p* < 0.01, **** *p* < 0.0001). Graphs are shown as mean ± SEM.

To further characterize the effect differences between the two glucose exposure settings, we directly compared the 30- and 25-mM glucose samples. A total of 314 DEPs (FC ≥ 1.5, *p* < 0.05) were found to be differentially regulated (Supplemental Table S1), with most of them presenting a downregulated pattern (Figure 4a). Moreover, just two out of 14 previously characterized β-cell constitutively expressed proteins (DDX17 (DEAD-Box Helicase 17), PPIB (Peptidylprolyl Isomerase B)) [35,36] were found differentially expressed (Supplemental Table S2). The pathway analyses of the overall proteome landscape pointed towards the deregulation of signaling involved in protein synthesis, antioxidant activity and cytoskeleton organization. As such, the EIF2 signaling pathway was detected as the top canonical pathway characterizing the analyzed landscape and inferred as inhibited (Figure 4b, blue rectangle). Consistently, the top rated network consisted of proteins involved in protein synthesis, with all but one being significantly downregulated in the samples exposed to 30 mM glucose, indicating that the highly elevated glucose concentration inhibits protein synthesis (Figure 4c).

Of note, one of the main detox pathways, the NRF2-mediated oxidative stress response (top two), was also predicted to be downregulated as compared to the 25-mM glucose condition, suggesting a lower capacity of the 30-mM-exposed cells to maintain the redox balance. This conclusion is also corroborated by the upstream analysis, which indicated NFE2L2 (Nuclear Factor, Erythroid 2 Like 2, NRF2) as one of the top inhibited upstream regulators of the analyzed proteome landscape (Figure 4d). The overview of the global proteome landscape also indicated a clear interplay between the inhibition of (1) NFE2L2/NRF2 (antioxidant activity), (2) β-catenin (canonical Wnt pathway) and (3) EIF2 signaling and EIF4 (protein synthesis) as the main fingerprint of the analyzed subset (Figure 4e, red arrows).

To validate this finding, we checked NRF2 activity by immunofluorescence and found a clear difference in the expression patterns between the two elevated glucose levels (Figure 4f). Following exposure to 25 mM ectopic glucose, the cells presented a dual cytoplasmic (inactive) and nuclear (active) pattern of NRF2 staining. In contrast, at 30 mM glucose, the pattern was exclusively cytoplasmic, indicating a lower antioxidant activity, in accordance with the pathway analysis.

In addition, superoxide dismutase 1 (SOD1), the main superoxide dismutase responsible for eliminating free superoxide radicals, was also significantly downregulated following exposure to 30 mM glucose (Figure 4g), further indicating a reduced antioxidant activity in these samples. Moreover, G6PC2, a key enzyme involved in glucose-6-phosphate hydrolysis in β-cells, was strongly downregulated after 30 mM glucose exposure, indicating increased glycolysis pathway activity in this glucose condition (Figure 4h). This, connected with antioxidant pathways in response to highly elevated glucose concentrations, suggests a potential unbalanced redox equilibrium in these samples.

### 2.7. Elevated Glucose Concentrations and Redox Imbalance Prompt Similar but not Overlapping Responses

**Figure 5:**
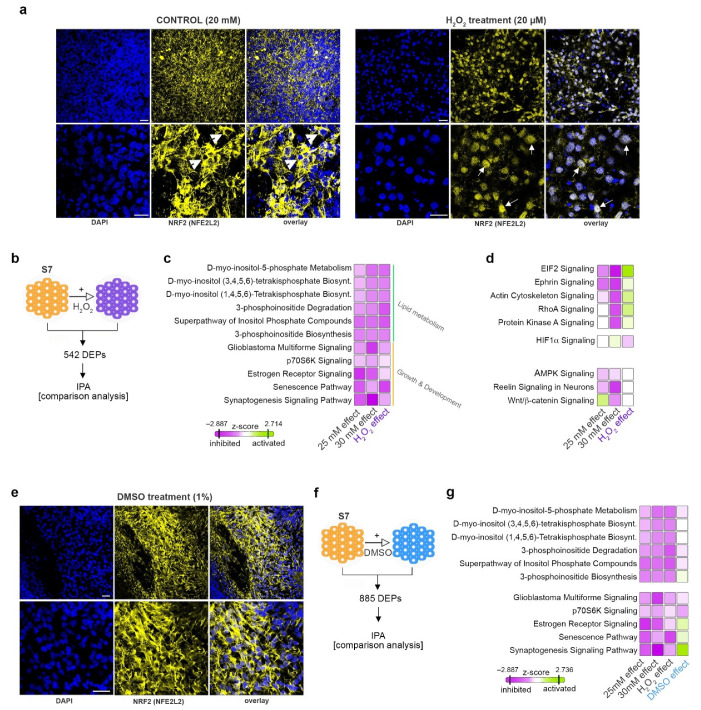
H_2_O_2_ and DMSO impact on differentiating pancreatic progenitors. (**a**) Confocal imaging of representative NRF2^+^ cells (yellow) in control (20 mM glucose) and H_2_O_2-_treated samples (Scale bar: 25 µm; blue–DAPI). (**b**) Scheme depicting the two conditions analyzed. (**c**) IPA-generated comparison analysis of the protein landscapes characterizing the 25 mM, 30 mM and H_2_O_2_ effects displaying similar or (**d**) dissimilar pathway regulation between the conditions analyzed. (**e**) Confocal imaging of representative NRF2^+^ cells (yellow) in control (20 mM glucose) and DMSO_-_treated samples (Scale bar: 25 µm; blue–DAPI). (**f**) Scheme depicting the conditions analyzed. (**g**) IPA-generated comparison analysis of the protein landscapes characterizing the 25 mM, 30 mM, H_2_O_2_ and DMSO effects.

To determine the contribution of a potential redox imbalance to the observed elevated glucose-induced effects, we treated the differentiating cells with H_2_O_2_, a known exogenous oxidative stress inducer, and analyzed their regulatory landscape using global TMT-plex proteomics. To validate the H_2_O_2_ effect on the redox balance and antioxidant activity, we checked the NRF2 expression level by immunofluorescence. We observed a clear difference between H_2_O_2_ treatment and the control (20 mM), with the peroxide-treated samples exhibiting a strong nuclear pattern on NRF2 (activated), while the control condition displayed a clearly cytoplasmic NRF2 staining. This result suggests that the H_2_O_2_-treated samples are compensating for the shift in redox balance by activating the antioxidant mechanisms (Figure 5a).

A total of 542 DEPs (Supplemental Table S1) were filtered between the H_2_O_2_-treated samples and the control untreated samples (Figure 5b). We further performed a pathway analysis comparison to match the resultant regulatory profile against the ones characterizing the response to elevated glucose concentration exposure. This revealed that both the H_2_O_2_-induced redox imbalance and the increased glucose level conditions are characterized by the decreased activity of the pathways involved in lipid metabolism (Figure 5c, green bracket). Moreover, key pathways regulating development and growth presented similar activity patterns (Figure 5c, orange bracket). In contrast, H_2_O_2_ exposure elicited an opposite effect compared to glucose exposure on pathways involved in protein synthesis (such as EIF2 signaling) or cellular remodeling and integrin signaling (RhoA and actin cytoskeleton signaling) (Figure 5d). Of note, key pathways regulated by elevated glucose concentrations, such as the canonical Wnt pathway, do not respond to H_2_O_2_ treatment, suggesting that these are not modulated by glucose via changes in the energy metabolism and redox balance, but rather through a different mechanism.

To validate the role of glucose-induced redox imbalance on modulating the subset of pathways affected by both glucose concentration and H_2_O_2_, the differentiating cells were treated with DMSO, a known antioxidant agent. Due to its antioxidant action, DMSO was expected to promote an opposite regulatory pattern on this group of pathways, given that they are indeed regulated by shifts in the redox balance. Indeed, assessing the NRF2 pattern by immunofluorescence revealed a strongly cytoplasmic staining, consistent with low oxidative stress as anticipated following antioxidant treatment (Figure 5e).

A total of 885 DEPs (Supplemental Table S1) were filtered between the DMSO-treated and control samples (Figure 5f) and the resultant landscape profile was compared against the ones characterizing the H_2_O_2_-, 25-mM and 30-mM glucose-exposed samples.

With the exception of 3-phosphoinositide biosynthesis signaling, the pathways involved in lipid metabolism were largely unaffected by the DMSO action (Figure 5g), suggesting that these are either irresponsive to antioxidant stimulation or that H_2_O_2_ and elevated glucose act through non-overlapping independent mechanisms.

In contrast, the DMSO treatment induced an opposite regulatory pattern in several pathways modulated by both H_2_O_2_ and elevated glucose concentrations; nevertheless, this effect seemed restricted to signaling involved in growth and development (such as estrogen signaling pathway, senescence pathway and synaptogenesis signaling pathway, Figure 5g).

## 3. Discussion

Here, we identified the in vitro proteome landscape changes characterizing hiPSC-derived islet cells exposed to elevated glucose levels from the pancreatic progenitor stage. Interestingly, we discovered that the differentiating cells respond differently to the two glucose concentrations used (25 or 30 mM) by modulating distinct pathways and molecular targets.

Admittedly, one of the limitations of this study is that it does not address whether the effects of elevated glucose concentrations impact the protein expression levels or cell populations’ size. Thus, we cannot properly establish if the increased glucose levels interfere with the protein abundance or cell fate acquisition and maintenance. Although it is beyond the scope of this study to unequivocally pinpoint the exact cellular mechanism, based on the unchanged fraction of insulin-expressing cells and insulin levels, we can speculate that, at least in the case of β-cell expressed markers, the probable scenario involves changes in the expression rather than the number of positive expressing cells. Nevertheless, this hypothesis should be rigorously addressed in future studies.

**Figure 6:**
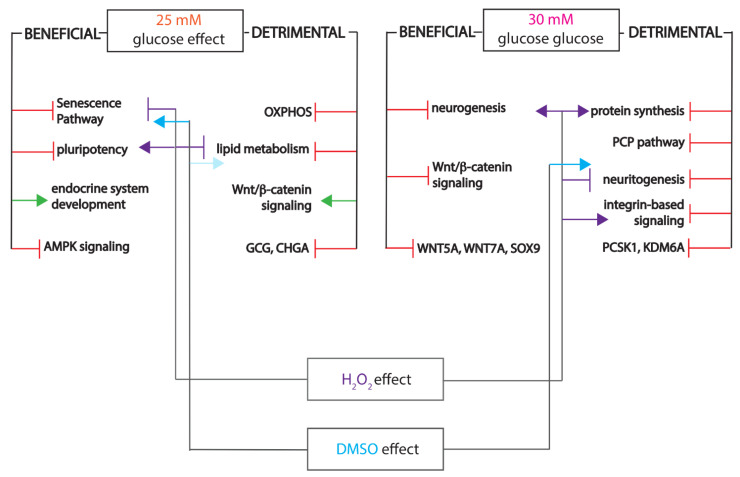
Integrative scheme depicting the beneficial and detrimental effects of elevated glucose levels, including superimposition of the H_2_O_2_ and DMSO treatments’ effects.

Of interest, the proteome landscape changes suggested both beneficial and detrimental changes for the islet cells fingerprint. Our data indicated that only a small subset of the identified regulatory landscape responds to changes in the redox balance, suggesting that not all glucose effects are elicited via shifts in energy metabolism (Figure 6).

One important aspect to consider is that the differentiating cells were exposed to elevated glucose levels for a prolonged period of time. Indeed, the increase in glucose concentration was performed starting with the pancreatic progenitor stage and it was maintained up until the end of the protocol—thus, for a total of two weeks of exposure. This is longer than the interval used in most other in vitro studies, which usually ranges from 24h to one week [11,37,38,39]. Thus, any acute or transient response to the increase in glucose concentration would not be captured by the analysis, which focused only on the long-lasting or incremental modifications. As such, the changes in the proteome landscape represent the result of two weeks of sustained, chronically elevated, glucose exposure. Nevertheless, it should be considered that we did not include an osmotic control, and hence, it cannot be distinguished whether the observed effect was caused exclusively by exposure to glucose or if the osmotic stress was also contributing factor.

Furthermore, our analysis focused on the global proteome changes in response to increased glucose levels and not on specific aspects of metabolism or development. This setup allowed for discriminating between the beneficial and the detrimental effects of exposure to elevated glucose levels. These antithetic effects were previously correlated mostly with the length of glucose exposure or glucose concentration. Indeed, studies showed that brief glucose treatments (≤24 h) could potentiate β-cell function [10,11], an effect also noticed in response to physiological glucose stimulation values (10 mM) [12]. In contrast, prolonged exposure or high glucose levels are detrimental for β-cell identity [40]. Nevertheless, our study shows that chronic high glucose exposure, besides the expected detrimental effects, also affects several proteome subsets in an apparently beneficial manner, one example being the inhibition of the canonical Wnt pathway. The importance of canonical and non-canonical Wnt pathways for hiPSC differentiation was already indicated by several studies [41]. A recent work [42] demonstrated the importance of canonical Wnt pathway inhibition during endocrine differentiation of hESCs, a result in agreement with our finding that inhibition of the Wnt/β-catenin pathway by highly elevated glucose concentration (30 mM) is beneficial for the islet signature of the differentiating cells.

Our analysis further indicated the inhibition of the Wnt/PCP pathway as a detrimental effect of the same glucose concentration, in line with the findings of [43], which uncovered that stimulating the non-canonical Wnt/PCP pathway by WNT5A upregulates β-cell maturation makers and improves differentiation in P5 islet cells and pseudo-islets of Min6 insulinoma cells. Of note, the regulation of the canonical Wnt/β-catenin pathway illustrates the differential effect elicited by the two glucose concentrations, being detrimentally activated by exposure to 25 mM glucose and beneficially inhibited by treatment with 30 mM. Nevertheless, the unequivocal link between canonical and non-canonical Wnt signaling and glucose levels absolutely requires further experimental evidence, which was not the object of this study. In addition, a direct comparison between the proteome landscapes evoked by the two elevated glucose concentrations also revealed differences in protein synthesis and antioxidant pathways, both inhibited in response to the highest glucose concentration. The reduction in the antioxidant activity represents an interesting result, as a large body of literature indicates [8,44,45] that glucose relays its effects by impacting the redox balance and triggering a signaling cascade, which ultimately results in an altered expression of key transcription regulators.

Of note, an additional aspect to consider here is glucotoxicity. The 30-mM concentration is a standard concentration at which glucose is expected to elicit deleterious effect on target tissues or cells. However, the pathway analysis of our datasets did not reveal typical signs of glucotoxicity, aside from the reduced levels of antioxidant activity, suggesting that the immature differentiating pancreatic endocrine cells cope differently with increased glucose concentrations than the mature ones.

Nevertheless, our comparative analysis revealed that only a small subset of pathways are collectively regulated by oxidant, antioxidant and chronic elevated glucose levels. Of interest, key developmental pathways regulating cell identity, such as the canonical and non-canonical Wnt pathways, were not part of this subgroup, being regulated only by chronic glucose exposure and not by peroxide or antioxidants (Figure 6). This observation can signal a potential redox-independent action of glucose. Alternatively, considering the length of elevated glucose exposure, the pathways refractory to redox imbalance can simply represent a secondary wave of regulation in response to the sustained impairment of energy metabolism. Moreover, it is possible that the immature energetic status of the in vitro differentiating cells alters the standard response to oxidant and antioxidant agents.

In this respect, one important drawback of our approach is that the control samples differentiated using the standard protocol were already exposed to very high, non-physiological glucose concentrations (20 mM). Thus, any impact on the redox balance already intrinsic to the control samples would not be revealed by the differential expression analysis. As such, the additional oxidative stress elicited by H_2_O_2_ treatment might have a low impact on an already redox-imbalanced environment caused by chronic glucose exposure. Regardless of explanation, the impact of chronic high glucose concentrations on key developmental pathways represents an important and exploitable finding.

Furthermore, the effects of the exogenous glucose exposure revealed here are in stark contrast with our in vivo observations. hiPSC-derived pancreatic progenitors generated by the same differentiation protocol and xenotransplanted into overtly hyperglycemic mice presented dysregulation of energy metabolism and redox balance as main profile changes [31]. This suggests that either the in vitro differentiating cells are too immature to mount a proper energy metabolism response to glucose induction, or that exposure to in vivo hyperglycemia is a more potent stimulus due to systemic action and interference from other organs.

Overall, these results support a dual impact of elevated glucose levels that vary according to concentration. Following chronic exposure, a large subset of the deregulated proteome landscape does not seem to be regulated by redox imbalance, in contrast with the in vivo exposure to hyperglycemia. Of interest, prolonged exposure to high glucose concentrations modulates pathways with a key role in islet cell identity. Once more, the targets and pathways revealed by this study require further experimental validation before an absolute conclusion can be reached regarding their modulation by high glucose concentrations.

## 4. Materials and Methods

### 4.1. Cell Sources and Ethical Statements

The experimental protocols reported in this study were approved by the Norwegian Regional Committee of Medical and Health Research Ethics for the use of hiPSCs (REK 2010/2295) and human islets (REK 2011/426). All methods reported here were carried out in accordance with the Helsinki Declaration. Informed consent was obtained from healthy donor (for skin biopsies) or from the relatives (for organ donations). The hiPSC lines characterized in this study were generated by episomal reprogramming using the following vectors obtained from Addgene: OCT3/4 (#27077), L-MYC, LIN28 (#27080) and SOX2, and KLF4 (#27078), as previously described by us [32,46]. The hiPS cells were checked to be negative for mycoplasma by using a MycoAlert Mycoplasm Detection Kit (Lonza, LT07-418) prior to differentiation. The pluripotency and differentiation potential of these iPSCs were previously assessed [32,33,46]. Human islets were obtained from the Juvenile Diabetes Research Foundation (JDRF) award 31-2008-416 (ECIT Islet for Basic Research program) and isolated as previously described [47] from males/female 2/1 non-diabetic brain dead donors with a mean age of 50 years (35–60 years) and a mean body mass index (BMI) of 25.7 kg/m^2^ (24–28 kg/m^2^), after receiving appropriate informed consent from relatives for multi-organ donation and for use in research. The human islet characteristics are presented in Table 1.

### 4.2. Cell Differentiation

Before in vitro differentiation, the hiPSC lines were enriched with stage-specific embryonic antigen-4 (SSEA4) positive cells by using magnetic beads (#130097855 MACS Miltenyi Biotec). Three different hiPSC lines were differentiated in independent experiments (differentiation rounds) according to a seven-stage protocol [24]. For this study, we used 2D differentiation on Matrigel-coated 6-well plates as previously described by us [33]. For each differentiation round, hiPSCs were cultivated in parallel dishes, with glass coverslips added to each well. At stage 5 (pancreatic endocrine precursors), differentiating cells were incubated in the respective differentiation media containing 3 different glucose concentrations respectively (standard, 20 mM; elevated, 25 and 30 mM). At stage 7, the cells were harvested for either global proteomics or immunofluorescence staining.

### 4.3. Cell Counting and Viability Measurements

For each experiment, the cell number and viability of each sample were measured using NucleoCounter NC-200 (ChemoMetec, Allerod, Denmark) following the manufacturer’s instructions for Via1-Cassette (cat. no. 941-0012) with Reagent A100 (cat. no. 910-0003) and B (cat. no. 910-0002).

### 4.4. H2O2 and DMSO Treatments

Differentiating hiPSC-derived cells (stage 7) were incubated in 20 µM hydrogen peroxide fresh solution (H1009-100ML, Sigma, St. Louis, MO, USA) or 1% Dimethyl Sulfoxide Solvent (DMSO D8418, Sigma, St. Louis, MO, USA) for 24 h and then harvested and processed further for global proteomics or immunofluorescence staining. Cell viability was above 85% in all conditions (Supplemental Figure S3).

### 4.5. Immunofluorescence Staining

Coverslips covered by differentiating hiPSC-derived cells were fixed in 2% Paraformaldehyde (PFA) for 20 min at room temperature, followed by several washes with phosphate-buffered saline (PBS). After blocking for 30 min at room temperature with 2% bovine serum albumin (BSA) in PBS, coverslips were incubated in primary antibody overnight at 4 °C. The following primary antibodies were used: guinea pig anti-insulin (1/500, A056401-2, Dako, Glostrup, Denmark) and rabbit anti-NRF2 (1/250, SAB4501984-100UG, Sigma-Aldrich, St. Louis, MO, USA,). After brief washes in PBS, the coverslips were incubated for 3 h at room temperature, in the dark, with the following secondary antibodies: goat anti-guinea pig A488 and donkey anti-rabbit A647 (1/500, Molecular Probes, Eugene, OR, USA). The nuclei were stained with DAPI (D1306, Molecular Probes, Eugene, OR, USA). The coverslips were mounted on glass slides using Prolong Diamond Antifade Mountant Media (P36970, Life technologies, Carlsbad, CA, USA) and images were acquired using a Leica TCS SP5 STED CW confocal microscope (Leica Microsystems, Wetzlar, Germany).

### 4.6. Global Proteomics Analysis

Differentiating cells at stage 7 and following respective treatments were washed in Dulbecco’s phosphate-buffered saline (DPBS, D8537, Sigma, St. Louis, MO, USA) and harvested with TrypLE Select Enzyme (1X) (12563011, Thermo Fisher Scientific, Waltham, MA, USA), followed by centrifugation. Cell pellets were lysed in a buffer of 8 M urea (U1250, Sigma, St. Louis, MO, USA), 200 mM EPPS (3-[4-(2-Hydroxyethyl)piperazin-1-yl]propane-1-sulfonic acid, E1894, Sigma, St. Louis, MO, USA) pH 8.5 and protease inhibitors (Roche Complete with Ethylenediaminetetraacetic acid (EDTA), Roche Basel, Switzerland, catalog number 11836153001) and sonicated for three rounds of 30 s at 30% power. Human islets were lysed in 4% SDS buffer and boiled at 95 °C for 7 min on a shaker, followed by sonication. The protein concentration was determined using a bicinchoninic acid (BCA) protein assay kit (Thermo Fisher Scientific, Waltham, MA, USA, catalog number 23225). Dry aliquots containing an estimated amount of 100 µg of proteins were further processed using the Filter-Aided Sample Preparation method. Tandem Mass Tag (TMT) 11-plex labeling, phase fractionation and LC-MS/MS analysis were performed at the Taplin Facility at Harvard Medical School as previously described [48]. The mass spectrometry proteomics data have been deposited to the ProteomeXchange Consortium (http://proteomecentral.proteomexchange.org, accessed on 11 February 2021) via the PRIDE partner repository [49] with the dataset identifier PXD022177.

### 4.7. Proteomic Data Analysis

We analyzed the mass spectrometry data as earlier described [31,32]. Hierarchical clustering on both entities and conditions using the squared Euclidian distance metric and Ward’s linkage rule was performed with GeneSpring 14.9.1 GX software (Agilent Technologies, Santa Clara, CA, USA). The pathway analyses were generated through the use of the Ingenuity Pathway Analysis Program (IPA, QIAGEN Inc., Redwood City, CA, USA) [50].

### 4.8. Statistical Analysis

Statistical analysis was performed using GraphPad Prism v8.4.3 (GraphPad Software Inc., La Jolla, CA, USA). The unpaired two-tailed Student’s t-test was used for direct group comparison. A one-way ANOVA with Tukey’s multiple corrections test was employed for multiple group comparison. In both cases, a *p*-value of < 0.05 was considered significant. In figures, data are represented as mean ± SEM.

## Figures and Tables

**Table 1:** Checklist for reporting human islet preparations used in research. Table format adapted from (Hart and Powers, 2019).

Islet Preparation	1	2	3
**Mandatory Information**			
Donor age (years)	60	35	57
Donor sex (male: M/female: F)	M	M	F
Donor BMI (kg/m^2^)	27.8	25.2	24.2
Donor HbA_1c_ or other measure of blood glucose control (mmol/mol)	44	not assessed	not assessed
Origin/source of islets	ECIT ^a^	EDIT	ECIT
Islet isolation centre	Oslo	Oslo	Oslo
Donor history of diabetes?	No	No	No
**Recommended Information**			
Donor cause of death	DBD ^b^	DBD	DBD
Warm ischemia time (h)	02:00	03:00	02:25
Cold ischemia time (h)	05:03	09:48	07:07
Estimated purity (%)	53	50	70
Estimated viability (%)	90	95	90
Total culture time (h) ^c^	72	72	72
Glucose-stimulated insulin secretion	2.3	2.2	4.3
Handpicked to purity?	Yes	Yes	Yes

a—European Consortium for Islet Transplantation. b—Donation After Brain Death. c—Time of islet culture at the isolation center, during shipment and at the receiving laboratory.

## Data Availability

The datasets generated and analyzed for this study can be found in the ProteomeXchange via the PRIDE partner repository with the dataset identifier PXD022177.

## Supplementary Materials

The following are available online at https://www.mdpi.com/article/10.3390/ijms22073698/s1, Figure S1: IPA-generated global integration of all pathway analysis levels characterizing the 25 mM glucose condition, Figure S2: IPA-generated global integration of all pathway analysis levels characterizing the 30 mM glucose condition, Figure S3: Cell viability at the end of stage 7 after DMSO and H2O2 treatments. Table S1: List of Differentially Expressed Proteins, Table S2: Differentially Expressed Beta-cell Housekeeping Proteins.
