# Supplementary material for: Chronically Elevated Exogenous Glucose Elicits Antipodal Effects on the Proteome Signature of Differentiating Human iPSC-Derived Pancreatic Progenitors
Source: Int J Mol Sci. 2021 Apr 2;22(7):3698. doi: 10.3390/ijms22073698 (PMC8038174; doi:10.3390/ijms22073698)
Supplement: Supplementary file 1 [file ijms-22-03698-s001.zip › ijms-1126391 sup resub.docx]

Supplementary Material

##
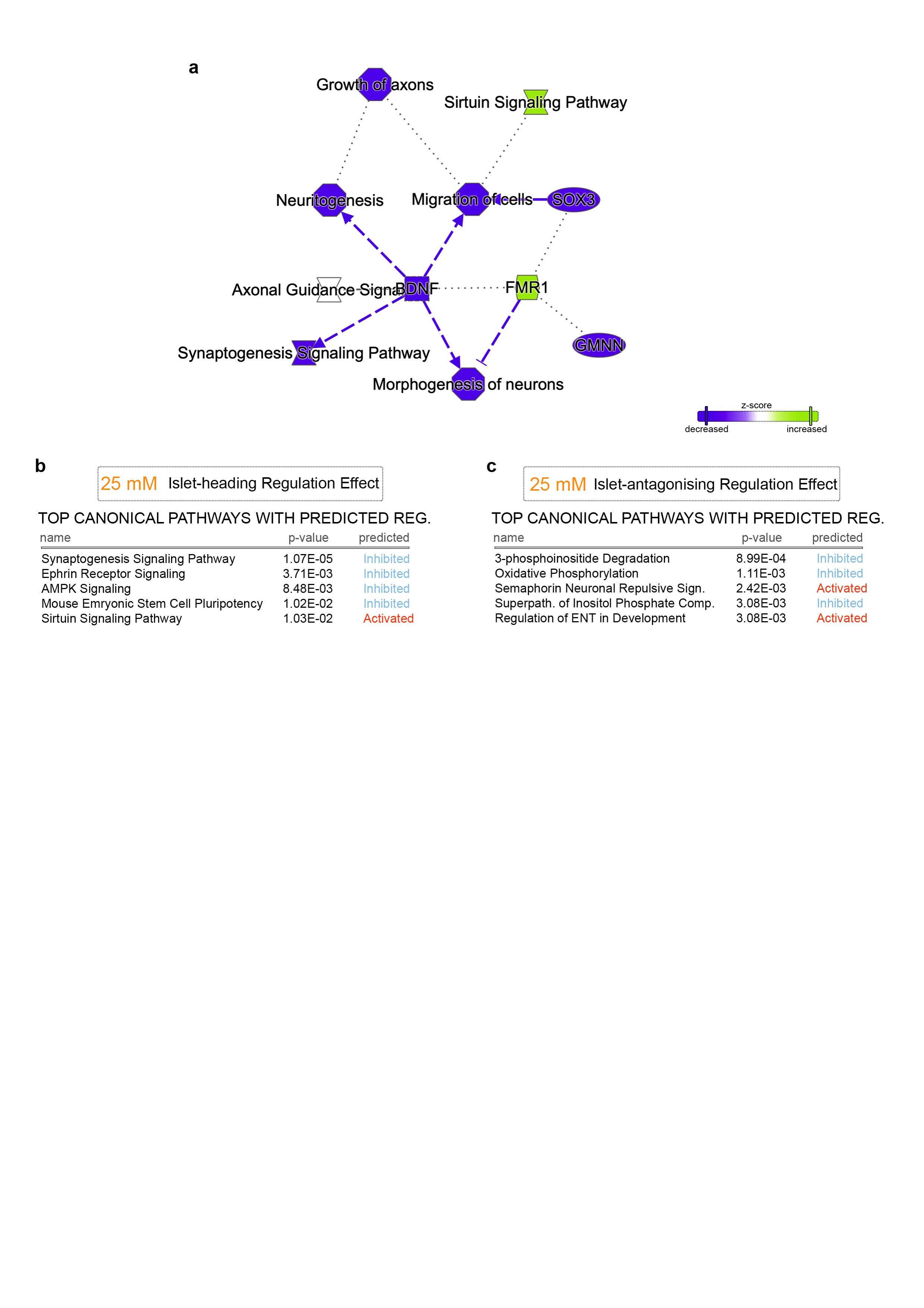


**Supplementary Figure 1.** (a) IPA generated global integration of all pathway analysis levels characterising the 25 mM glucose condition. (b) Top canonical pathways with predicted regulation characterizing the subset of proteins with islet-heading regulatory pattern following 25 mM glucose exposure. (c) Top canonical pathways with predicted regulation characterizing the subset of proteins with islet-antagonising regulatory pattern following 25 mM glucose exposure.

**
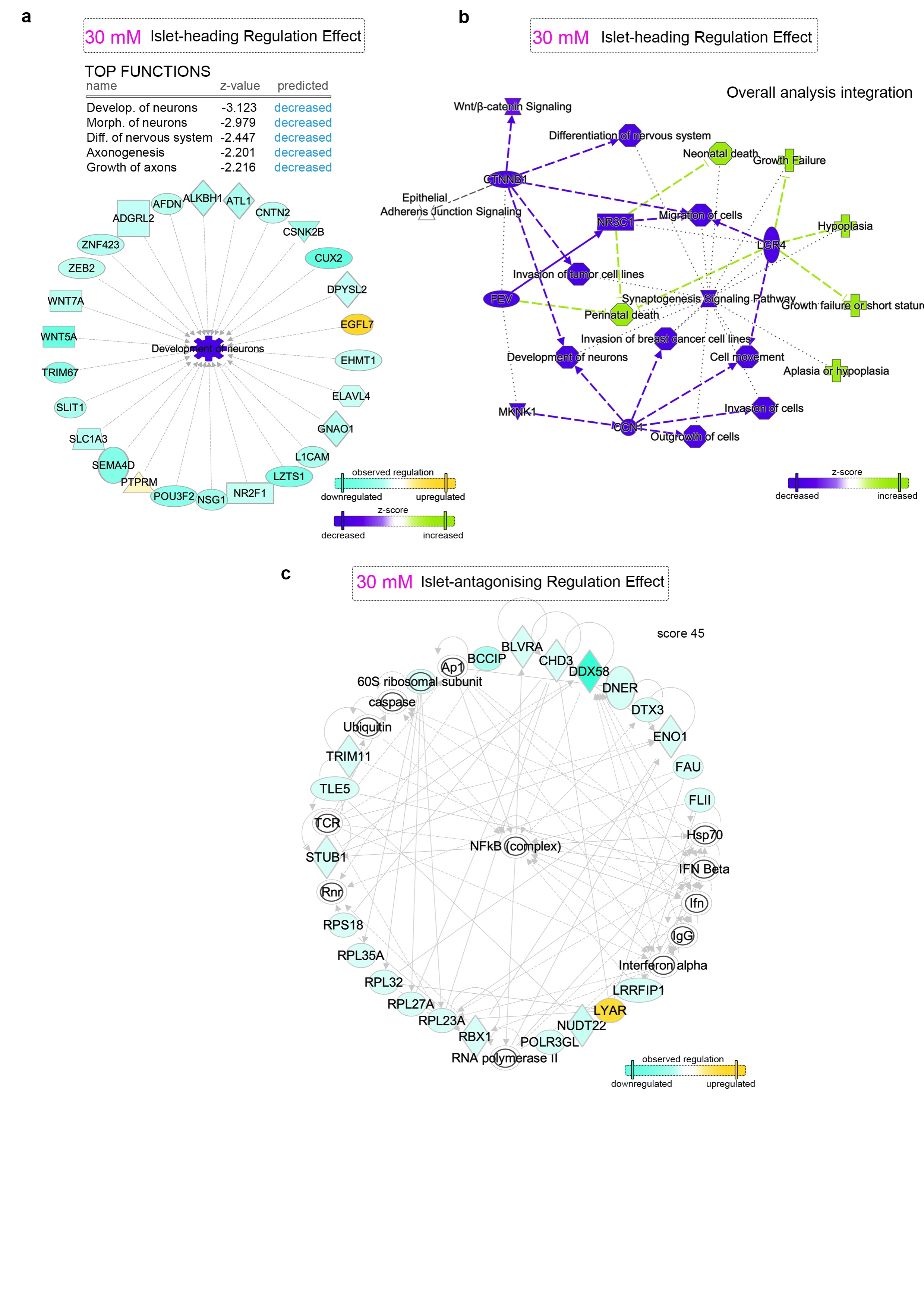
**

**Supplementary Figure 2.** (a) IPA-generated disease and function top and selected network characterizing the top disease and function processes. (b) IPA generated global integration of all pathway analysis’ levels characterising the 30 mM glucose condition. (c) Selected top radial network characterising the protein subgroup with islet-antagonising regulatory pattern after 30 mM glucose exposure, displaying the observed downregulation of proteins involved in protein synthesis.

**
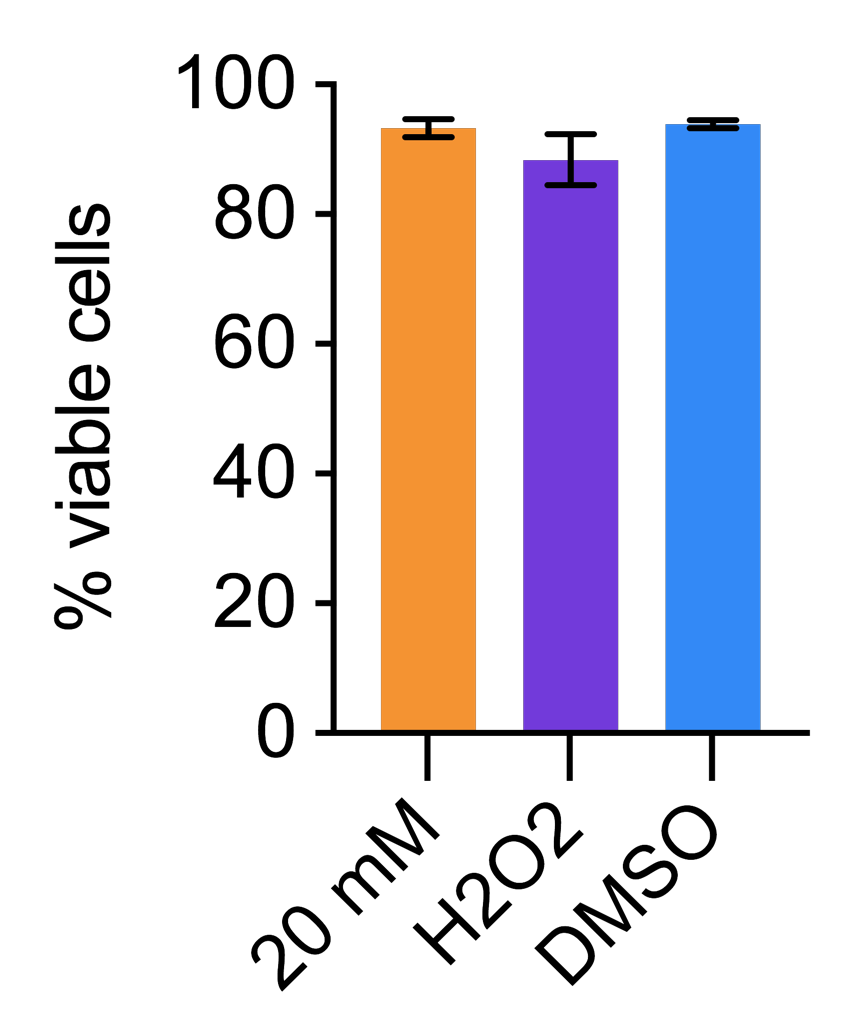
**

**Supplementary Figure 3.** Cell viability at the end of stage 7 after treatments with DMSO or H_2_O_2_.

**Supplementary Table 2.** Differentially Expressed Beta-cell housekeeping proteins

| Differentially Expressed Beta-cell Housekeeping Proteins | | | |
| --- | --- | --- | --- |
| (30 mM *versus* 25 mM) | | |  |
| DDX17 | | | |
| PPIB | | | |
|  |  |  |  |
| Differentially Expressed Beta-cell Housekeeping Proteins | | | |
| (30 mM *versus* Standard Differentiated Stage 7 cells) | | | |
| none | | | |
|  |  |  |  |
| Differentially Expressed Beta-cell Housekeeping Proteins | | | |
| (25 mM *versus* Standard Differentiated Stage 7 cells) | | | |
| VAMP2 | | | |
